# Universal differential equations for quantifying NF-κB—p53 signaling crosstalk

**DOI:** 10.1093/bioinformatics/btag449

**Published:** 2026-08-21

**Authors:** Umur Can Kaya, Xhemal Kodragjini, Samuel Zambrano, Katharina Baum

**Affiliations:** Department of Mathematics and Computer Science, Freie Universität Berlin, Berlin 14195, Germany; Hasso-Plattner-Institute, Digital Engineering Faculty, University of Potsdam, Brandenburg 14482, Germany; Department of Mathematics and Computer Science, Freie Universität Berlin, Berlin 14195, Germany; Università Vita-Salute San Raffaele, Milan 20132, Italy; Division of Genetics and Cell Biology, IRCCS Ospedale San Raffaele, Milan 20132, Italy; Department of Mathematics and Computer Science, Freie Universität Berlin, Berlin 14195, Germany; Hasso-Plattner-Institute, Digital Engineering Faculty, University of Potsdam, Brandenburg 14482, Germany

## Abstract

**Summary:**

Universal differential equations (UDEs) have emerged as a powerful tool for scientific discovery, uniting differential equations and deep learning. Yet, their application to the noisy and complex data of systems biology remains limited. Here, we demonstrate that UDEs, paired with symbolic regression, can quantify the crosstalk between NF-κB and p53 signaling pathways based on experimental data exhibiting complex, oscillatory dynamics. We validate the framework on synthetic benchmarks, showing that crosstalk recovery is robust to measurement noise and improves markedly with the number of available time series. We then apply the framework to 106 simultaneously measured single-cell p53 and NF-κB time series following DNA damage and NF-κB activation, constituting the largest-scale UDE application in systems biology so far. UDEs with both a detailed and a minimal mechanistic p53 model consistently identify a monotonically increasing crosstalk function in which elevated NF-κB levels enhance p53 synthesis. Symbolic regression distills the learned neural network output into a compact, interpretable closed-form expression, providing a quantitative, time-resolved characterization of NF-κB-driven amplification of p53 at the single-cell level. Our work thus delivers a dual contribution: methodologically, it establishes UDEs as a viable tool for gaining quantitative insights in complex, noisy biological systems at scale; biologically, it provides a data-driven characterization of NF-κB-p53 pathway crosstalk from single-cell dynamics.

**Availability and implementation:**

Source code is available at: https://github.com/DILiS-lab/ude-crosstalk-discovery.

## 1 Introduction

Mathematical models are essential for understanding complex biological systems, yet constructing them remains difficult when our mechanistic knowledge is incomplete (Kitano 2002). Ordinary differential equation (ODE) models are the standard tool for this purpose (Klipp *et al*. 2005), but they require all relevant interactions to be specified in advance, a requirement that is rarely met in practice. When important processes are missing, parameter estimation may compensate by distorting fitted values, yielding models that reproduce the data without reflecting the true biological processes (Gutenkunst *et al*. 2007). The traditional remedy is hypothesis-driven model selection (Vyshemirsky and Girolami 2008, Kirk *et al*. 2013), but this scales poorly with the number of candidate mechanisms and is limited by what the modeler can anticipate.

Universal differential equations (UDEs) offer an alternative. Building on neural ODEs (Chen *et al*. 2018), Rackauckas *et al*. (2020) proposed replacing unknown terms in a mechanistic ODE with neural network components that learn the missing interactions directly from data. The known biological mechanisms are preserved while neural network components bridge the gaps, enhancing the model’s expressivity without sacrificing its underlying interpretability. Combined with symbolic regression (Cranmer *et al*. 2020, Rackauckas *et al*. 2020), the learned interaction functions can be distilled into closed-form expressions, turning black-box approximations into candidate mechanistic laws, enabling the formulation of new testable hypotheses. UDEs have gained traction across several disciplines due to their ability to quantify hidden relationships, with applications including geoscience (Bolibar *et al*. 2023), physics (Kuzhiyil *et al*. 2024), neuroscience (El-Gazzar and van Gerven, 2025), and systems biology (Philipps *et al*. 2025).

The UDE framework has been benchmarked on a range of biological systems using synthetic data, in an early anticipation of the UDE paradigm (Oliveira, 2004), for establishing identifiability analysis (Giampiccolo *et al*. 2024), for uncertainty estimation (Schmid *et al*. 2025), or for assessing regularization strategies (Philipps *et al*. 2025, de Rooij *et al*. 2025a). Applications to real biological data have so far been constrained to small mechanistic models and sparse measurement regimes: a 2-variable glucose model trained on a single time series of 7 time points (de Rooij *et al*. 2025a); conditional UDEs for inter-individual variability across 117 time series with 5 time points each (de Rooij *et al.* 2025b); bioeconomics models with at most three variables trained on single time series of 50–70 observations (Buckner *et al*. 2026); a 3-variable SIR model trained on 22 time points (Kuwahara and Bauch 2024); and an 8-variable STAT5 dimerization model, the most complex to date, trained on a single dataset of 16 time points across 3 observables (Philipps *et al*. 2024, 2025). Notably, all of these applications involve simple dynamics with at most one or two peaks per time series. Whether UDEs can move beyond parameter estimation to gain quantitative insights when applied to systems with more complex dynamics remains an open question.

**Figure 1:**
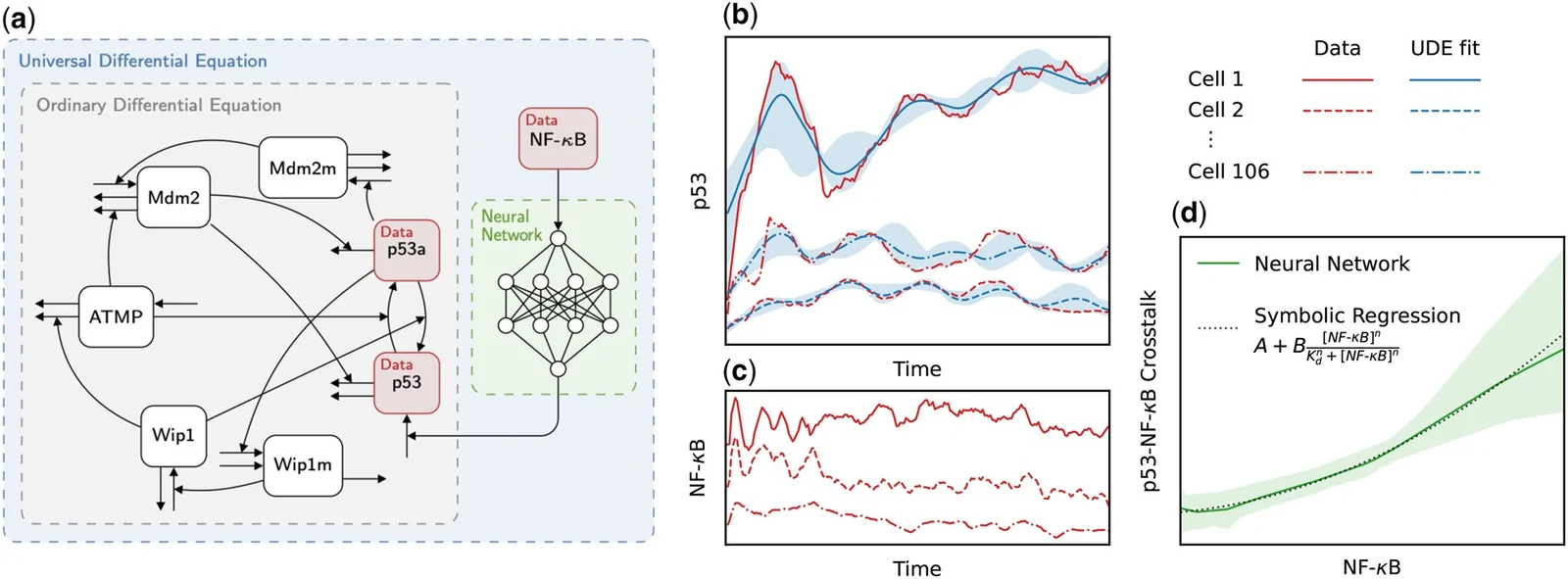
Overview of the UDE-based crosstalk discovery. (a) A mechanistic p53 ODE model (gray) is augmented with a neural network (green) that uses the measured NF-κB signal to modulate p53 production rate, forming a UDE model (blue). (b–d) The model is trained on experimental single-cell p53 and NF-κB time series (b–c, red, mock data). The fitted UDE model explains the p53 dynamics (b, blue curves) and learns the hidden crosstalk function (d, green), which is then distilled into an interpretable closed-form expression via symbolic regression (dotted).

In this work, we apply the UDE framework to quantify the crosstalk between the NF-κB and p53 signaling pathways from real experimental data (see Fig. 1). With their oscillatory response to stimuli, p53 and NF-κB signaling are examples for decisively more complex dynamics. p53 and NF-κB are two of the central regulators of the cells’ response to stress with high relevance to cancer research (Wang *et al*. 2023). Their interaction, or crosstalk, is highly relevant: p53 is the guardian of the genome, activated by a plethora of genotoxic stress signals and mutated frequently in cancer. Its activation dynamics is considered fundamental in determining cell fate decisions (Batchelor *et al*. 2009). On the other hand, NF-κB is a key player in response to inflammatory stimuli (Kizilirmak *et al*. 2022). The established link between inflammation and cancer makes understanding how NF-κB affects p53 activation an important question in cancer biology. Due to the association of p53 dynamics with cell cycle arrest or cell death, and of NF-κB with pro-proliferation signals, these two transcription factors have long been considered antagonists (Ak and Levine 2010). However, Konrath *et al.* (2020) suggested multiple conflicting mechanistic options for NF-κB’s effect on p53, comprising increased or decreased p53 degradation, in addition to other required alterations. Further, recently, we contributed to the development of a quantitative experimental framework to address the crosstalk, suggesting that NF-κB transcriptionally contributes to the up-regulation of p53 through increased *TP53* expression (Colombo *et al*. 2026). However, even this careful experimental analysis does not provide a complete quantitative characterization of how NF-κB amplifies p53.

Lacking temporal data of NF-κB, previous quantitative investigations of NF-κB-p53 crosstalk focused on estimation of time-independent perturbations in kinetic parameters on p53 data (Konrath *et al.* 2020). In contrast, our UDE approach incorporates the dynamic NF-κB signal over time. The dataset used for our analysis, generated and available from Colombo *et al*. (2026), comprises 106 simultaneously measured single-cell NF-κB and p53 time series of 200 time points following DNA damage and NF-κB stimulation, modeled using mechanistic ODE systems with up to 7 variables. Unlike the smooth, simple time series used in previous UDE studies, p53 exhibits complex multi-pulse oscillatory dynamics that vary substantially across individual cells. This complexity constrains the range of values of kinetic parameters suitable to fit the data. In addition, inferring a common crosstalk mechanism over not only a few but 106 independent cells, each oscillating differently, constrains the solution space further. Together, this enables the framework to move beyond model calibration toward genuine mechanistic discovery: learning a previously unknown crosstalk function directly from noisy single-cell measurements, and distilling it into an interpretable analytical expression via symbolic regression. Before applying the UDE framework to measured data, we perform systematic synthetic experiments mimicking our experimental conditions to establish whether it can reliably recover unknown crosstalk functions, and how recovery quality depends on crosstalk strength and type, noise level, number of available time series, and model complexity. Thus, our work provides a framework for the quantitative identification of crosstalk mechanisms between signaling pathways from single-cell dynamics data.

## 2 Methods

### 2.1 Experimental p53 and NF-κB data

**Figure 2:**
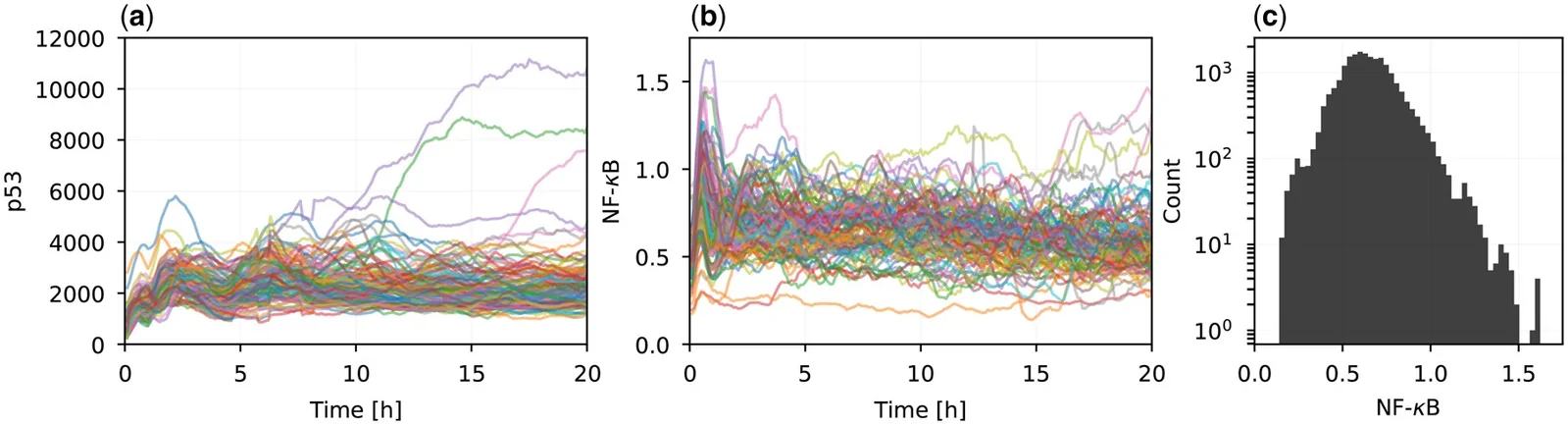
Experimental single-cell time series data. (a) p53 trajectories of 106 individual cells measured every 6 min over 20 h following DNA damage and NF-κB stimulation. (b) Corresponding NF-κB trajectories. (c) Distribution of NF-κB values across all cells and time points.

The data used for our analysis was generated in Colombo *et al*. (2026) via live cell imaging experiments. Images of MCF-7 cells expressing fluorescently tagged versions of p53 and NF-κB were recorded every 6 min for 20 h. We consider the dataset where a double stimulus, γ irradiation and TNF-α (which activate p53 and NF-κB, respectively), was applied, consisting of paired time series of 106 cells in total (see Fig. 2, Supplement 1, available as supplementary data at *Bioinformatics* online). Following other studies, the p53 time series correspond to background-corrected mean intensity nuclear levels of the fluorescently tagged p53; NF-κB time series correspond to the nuclear to cytosolic ratio of the fluorescence average intensity, a usual proxy for NF-κB nuclear concentration (Kizilirmak *et al*. 2022).

### 2.2 Mechanistic p53 ODE models

We use two mechanistic p53 models of different complexity. The Hunziker model (Hunziker *et al.* 2010) focuses on the p53-Mdm2 feedback loop with 4 variables and 9 kinetic parameters (see Supplement 2, available as supplementary data at *Bioinformatics* online). The Konrath model (Konrath *et al.* 2020), originally proposed for investigating crosstalk mechanisms to NF-κB via time-independent changes in kinetic rates, provides a more detailed representation with 7 variables and 24 kinetic parameters explicitly modeling the feedback regulators Mdm2 and Wip1 and the upstream kinase ATM (see Supplement 3, available as supplementary data at *Bioinformatics* online). For both models, cell-specific measurement parameters $a_{m}$ (scaling) and $b_{m}$ (offset) map simulated p53 concentrations to fluorescence units: ${\hat{y}}_{m}(t)=a_{m}\cdot x_{p53,m}(t)+b_{m}$, where $x_{p53,m}$ denotes nuclear p53 in the Hunziker model and the sum of active and inactive p53 in the Konrath model.

### 2.3 Universal differential equations

The general form of our UDE model is given by


$$\frac{dx}{dt}=f(x,p,U_{\theta }(z(t))),$$

where x represents the variables of the ODE system. The function *f* characterizes the system’s dynamics, governed by kinetic parameters p and augmented by a neural network component $U_{\theta }$. This network, parameterized by learnable weights θ, approximates the unknown function describing the exact crosstalk between p53 and NF-κB by taking the external NF-κB signal z(t) as input.

#### 2.3.1 Hybrid NF-κB–p53 crosstalk model

New experimental evidence supports that NF-κB activation leads to increased transcription of *TP53*, the gene encoding p53 (Colombo *et al*. 2026). Specifically, *TP53* expression was increased upon inflammatory signals, but less so if the p65 subunit of NF-κB was knocked out. Therefore, we parsimoniously assume that only the p53 synthesis rate varies upon changes in NF-κB. That this can lead to the experimentally observed shift in p53 dynamics was shown by qualitative modeling (Colombo *et al*. 2026), but the specific quantitative dependence of this increased rate on NF-κB was not inferred. Consequently, in our UDE framework, we approximate the crosstalk function with the neural network (NN) $U_{\theta }$ that acts as a modulator of the p53 synthesis rate:


(1) $$s_{p53-\text{eff}}(z(t))=s_{p53}\cdot U_{\theta }(z(t))$$

The NN receives the scalar NF-κB signal z(t), obtained via linear interpolation of the measured time series, and outputs a non-negative scaling factor that modifies the basal synthesis rate to obtain the effective p53 production rate $s_{p53-\text{eff}}$. Full details on the multi-layer perceptron architecture are provided in Supplement 5, available as supplementary data at *Bioinformatics* online.

#### 2.3.2 Constrained optimization

We employ a multi-level optimization strategy to account for cell-to-cell variability while identifying a universal crosstalk mechanism. The NN weights θ are shared across all single-cell time series, representing the common biological signal processing logic. In contrast, the kinetic parameters $p_{m}$, initial conditions $x_{0,m}$, and measurement parameters $a_{m}$ and $b_{m}$ are estimated individually for each cell *m* (m=1,…,M, where *M* is the total number of single cells in the dataset). This allows the model to fit distinct kinetic parameters for every cell, ensuring that the NN captures the true underlying crosstalk function rather than compensating for parameter heterogeneity.

The complete parameter set $\Phi =\{\theta \}\cup {\left\{p_{m},x_{0,m},a_{m},b_{m}\right\}}_{m=1}^{M}$ is estimated by minimizing the mean Huber loss (Huber 1964) over mini-batches:


$$L(\Phi )=\frac{1}{B\cdot K}\sum_{m=1}^{B}\sum_{k=1}^{K}L_{\delta }(y_{m,k}-{\hat{y}}_{m}(t_{k},z_{k};\Phi )),$$

where *B* is the batch size, *K* the number of time points, $y_{m,k}$ the measured p53 signal, ${\hat{y}}_{m}(t_{k},z_{k};\Phi )$ the p53 model prediction using cell-specific parameters, and $z_{k}$ the NF-κB signal at time $t_{k}$. The Huber loss transitions from quadratic to linear for residuals exceeding δ=1, providing robustness against outliers.

To ensure biologically plausible parameter values during training, the kinetic parameters $p_{m}$ and initial conditions $x_{0,m}$ are constrained to strictly positive ranges via a sigmoid reparameterization, with bounds defined relative to published nominal values (Hunziker *et al.* 2010, Konrath *et al.* 2020). The kinetic parameters are initialized by sampling from a log-normal distribution centered on these nominal values, providing diverse starting points for ensemble runs (Section 2.3.3). Initial conditions are obtained through a pre-equilibration phase of the unstimulated ODE system, and measurement parameters $a_{m}$ and $b_{m}$ are initialized to global constants. Full details on the implementation, reparameterization, bound construction and initialization procedures are provided in the Supplement.

#### 2.3.3 Uncertainty quantification

Following Schmid *et al*. (2025), we adopt an ensemble approach to quantify uncertainty. We perform $N_{\text{ens}}=10$ independent training runs, each with a unique random seed that diversifies the initialization of NN weights and kinetic parameters, as well as the mini-batch ordering during optimization. As is common in high-dimensional nonlinear parameter estimation (Gutenkunst *et al*. 2007, Westny *et al*. 2024), a minority of ensemble members exhibit diverging dynamics and are excluded (see Supplement 7, available as supplementary data at *Bioinformatics* online), yielding a final ensemble size of $N_{\text{ens}}\geq 8$ per experiment. The crosstalk function estimate is given by the ensemble mean, with the variance across members providing a measure of uncertainty.

### 2.4 Symbolic regression

To obtain an interpretable closed-form approximation of the quantified crosstalk function, we apply symbolic regression post hoc to the ensemble mean of the learned NN output $U_{\theta }(z)$. We apply weighting by the inverse variance of the ensemble predictions so that regions of high ensemble agreement contribute more to the fit (see Supplement 9, available as supplementary data at *Bioinformatics* online).

### 2.5 Synthetic data experiments

To validate the UDE framework and its ability to recover unknown crosstalk functions, we conduct synthetic data experiments where the ground-truth crosstalk function is predefined. Synthetic NF-κB time series are generated by resampling and augmenting real experimental signals to preserve biological noise and temporal characteristics. Synthetic p53 time series are then generated by simulating the Hunziker and Konrath ODE models with a known crosstalk function applied to the p53 synthesis rate via Equation 1. We define four ground-truth crosstalk functions based on weakly or strongly activating or inhibitory Hill-type kinetics (see Supplement 10, available as supplementary data at *Bioinformatics* online). To assess data requirements and noise robustness, we vary the number of single-cell time series M∈{16,128,512} and inject multiplicative log-normal measurement noise at three levels η∈{0.001,0.01,0.1} (see Supplement 11, available as supplementary data at *Bioinformatics* online for details).

## 3 Results

### 3.1 Experiments on synthetic data

**Figure 3:**
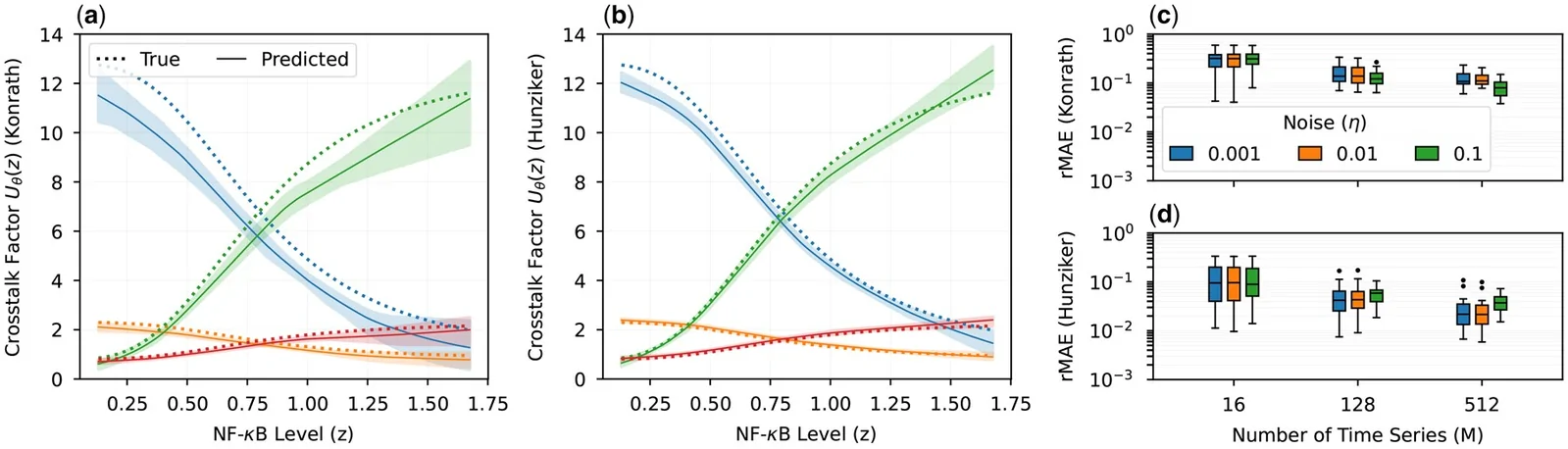
Synthetic data experiments for crosstalk recovery. (a, b) Learned crosstalk as a function of NF-κB for the (a) Konrath and (b) Hunziker based UDE models, evaluated on synthetic data containing M=128 time series with noise level η=0.1. Solid lines represent the ensemble mean with 5th–95th percentile shaded intervals. Dotted lines show the ground-truth crosstalk function. (c, d) rMAE of the recovered crosstalk for the (c) Konrath and (d) Hunziker based UDE models as a function of the number of training time series *M* and noise level η. Each box aggregates rMAE values across all four crosstalk function shapes and ensemble members.

We first validate the UDE framework on synthetic data where the ground-truth crosstalk function is known, allowing us to assess recovery accuracy in a controlled setting before applying the method to real experimental data. Both the more complex Konrath and less complex Hunziker based UDE models, trained on 128 single-cell time series at the highest noise level (η = 0.1), successfully recover all four ground-truth crosstalk functions (strong and weak activating, strong and weak inhibitory, Fig. 3a and b). This demonstrates that the framework is not restricted to a particular functional form but can recover both directions and magnitudes of crosstalk. The median relative mean absolute errors (rMAE, see Supplement 12, available as supplementary data at *Bioinformatics* online) range between 1% and 30% across function types and ensemble members. The ensemble uncertainty, reflected in the shaded 5th–95th percentile bands, is small across most of the NF-κB signal range and increases only at lower and higher NF-κB values where data density is low (see Supplement 11, available as supplementary data at *Bioinformatics* online). This widening is most pronounced for the strong activating function (Fig. 3a and b green), where the crosstalk factor takes on its most extreme values in precisely the data-sparse region.

Recovery quality improves with the number of available time series (Fig. 3c and d). Across all four crosstalk shapes and ensemble members, the largest improvement occurs between M=16 and M=128, with a smaller gain at M=512, suggesting that approximately 128 time series are sufficient for reliable recovery under these conditions. Overall, the Hunziker-UDE achieves better recovery than the Konrath-UDE in all settings (mean rMAE of 0.073 vs. 0.188). This is consistent with the expectation that crosstalk is easier to identify in a less complex underlying system, where fewer confounding interactions compete with the NN for explaining the data.

Across all sample sizes, the three noise levels yield largely overlapping rMAE distributions, indicating that the UDE framework is widely robust to measurement noise within the tested range and that sample size *M* is the dominant factor governing recovery quality. A breakdown by crosstalk function type (Supplement 13, available as supplementary data at *Bioinformatics* online) reveals that noise has opposite effects depending on signal strength: recovery of strong activating and inhibitory functions slightly improves at higher noise, while recovery of weak functions deteriorates, probably as noise masks the smaller signal amplitude.

### 3.2 Crosstalk quantification from measured time series

**Figure 4:**
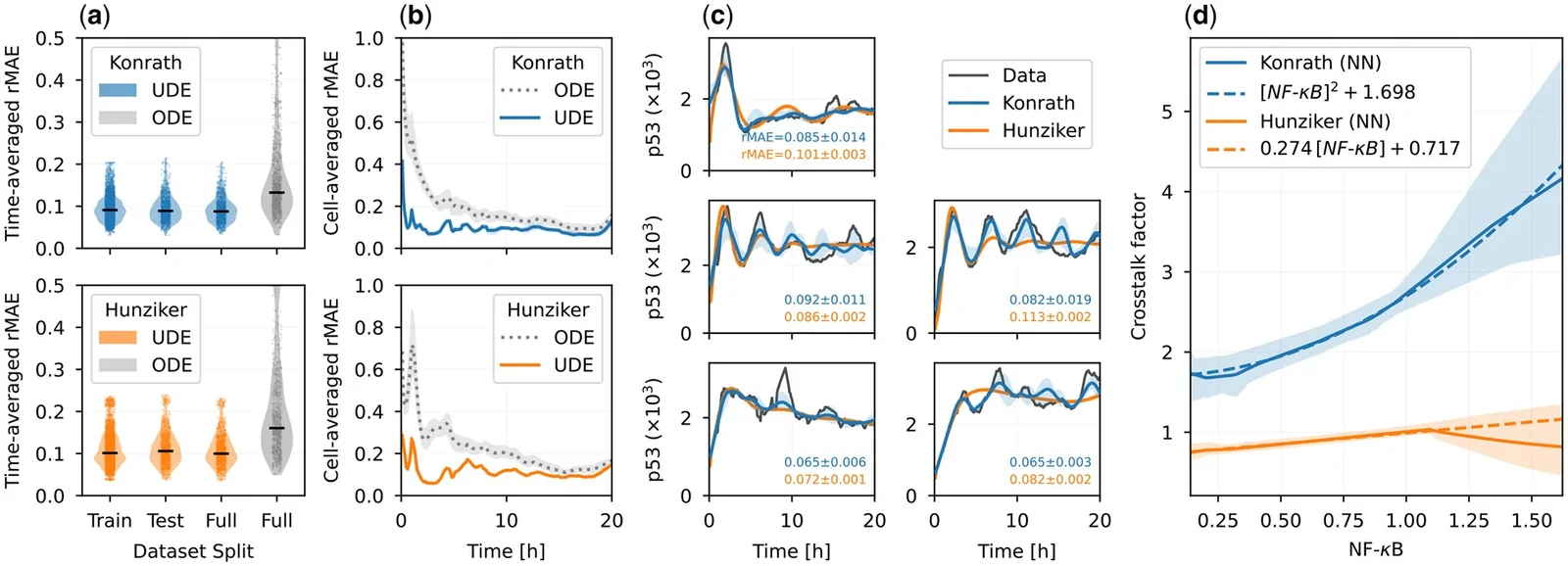
UDE-based quantification of NF-κB-p53 crosstalk from experimental single-cell time series data. (a) Distribution of time-averaged rMAE of p53 of individual cells for the Konrath (top) and Hunziker (bottom) UDE models evaluated on their held-out test cells (CV test) and training cells (CV train) from a 5-fold cross-validation experiment, the full-data UDE evaluated on all cells, and the full-data ODE baseline. (b) Cell-averaged rMAE of p53 per time point for the Konrath-based (top) and Hunziker-based (bottom) UDE models compared to the respective ODE baselines without crosstalk. (c) Representative fits of the Konrath-based and Hunziker-based UDE models to randomly selected p53 single-cell time series with per-cell rMAE ± ensemble standard deviation. (d) Recovered crosstalk functions $U_{\theta }$ for the Konrath and Hunziker UDE models and their symbolic regression approximations. Plots in (b–d) show the ensemble mean with 5th-95th percentile shaded intervals.

We apply the validated UDE framework to simultaneously measured single-cell NF-κB and p53 time series following DNA damage and TNF-α stimulation. To assess whether incorporating a crosstalk term is meaningful, we fit both a baseline p53 ODE model (without crosstalk) and the full UDE model to the data for each of the two mechanistic p53 models. We first conduct a 5-fold cross-validation (by cells) to rule out overfitting. The learned crosstalk function transfers to held-out cells: test and training rMAE for p53 are indistinguishable (Fig. 4a), and the crosstalk function’s shape is stable across folds, matching the full-data fit (Supplement 8, available as supplementary data at *Bioinformatics* online). We therefore use the full-data fit, which allows optimal crosstalk quantification. Both UDE models achieve a substantially lower rMAE compared to their respective ODE baselines throughout the 20-hour observation window (Fig. 4a and b). For the Konrath-UDE, the UDE reduces the mean rMAE in p53 from 0.188±0.016 to 0.093±0.002. Notably, the reduction in ensemble variance indicates that introducing the crosstalk term not only improves fit quality but also leads to a more consistent solution across all ensemble members. Overall, kinetic parameter distributions are highly similar between the UDE and ODE model (Supplement 14, available as supplementary data at *Bioinformatics* online). Only the parameter that characterizes p53 synthesis ($\beta_{p}$) is shifted to higher values in the baseline ODE model without crosstalk, compensating the lack of an NF-κB-signal mediating crosstalk function in this reaction. For the Hunziker-UDE, the UDE similarly reduces the mean rMAE from 0.224±0.018 to 0.113±0.001, with comparable gains in ensemble stability. However, no differences in kinetic parameter distributions can be observed, suggesting a reduced impact of the crosstalk in this model (Supplement 14, available as supplementary data at *Bioinformatics* online). Together, these results demonstrate that assuming a crosstalk between NF-κB and p53 is not only justified but substantially improves the mechanistic model’s ability to describe the data.

For both the Hunziker and Konrath-based UDE model, distributions of estimated kinetic parameters deviate slightly from their initialized values but still cover biologically meaningful ranges, indicating a successful constrained optimization. This is also visible in the fits to randomly selected single cell p53 time series for the UDE models that largely capture the observed dynamics (Fig. 4c). Overall, as suggested already by the lower mean rMAE, the Konrath-based UDE tends to provide more suitable fits, probably due to its higher complexity.

Both models identify a monotonically increasing crosstalk function, indicating that higher NF-κB levels are associated with enhanced p53 synthesis (Fig. 4d). However, the two models differ substantially in the strength of the inferred effect. The Konrath-based UDE learns a strongly increasing function, rising from a crosstalk factor of ∼1.8 to ∼4.5 across the observed range. In contrast, the Hunziker-based UDE identifies a much weaker effect, with the crosstalk factor remaining nearly flat between 0.8 and 1.1. In both cases, ensemble uncertainty widens substantially above NF-κB ≈1.1, consistent with the sparse data coverage at high NF-κB levels (see Fig. 2c).

Applying symbolic regression to the ensemble mean crosstalk function yields a quadratic expression $\hat{U}(z)=1.698+1\cdot z^{2}$ for the Konrath-UDE and a linear one $\hat{U}(z)=0.717+0.274\cdot z$ for the Hunziker based model (Fig. 4d). However, experimental data suggest that crosstalk is mediated by transcription, a gene regulatory process in which it is reasonable to expect a nonlinear dependence on NF-κB through Hill-type functions, $c_{\text{off}}+c_{\text{sc}}\cdot \frac{z^{n}}{K_{d}^{n}+z^{n}}$, as the one we used in our synthetic data validation. Fitting a Hill-type function independently to the ensemble mean crosstalk function of the Konrath-based UDE yields a Hill coefficient of n=2.114 and a Hill constant of $K_{d}=3.245$ (see Supplement 15, available as supplementary data at *Bioinformatics* online). Because $K_{d}$ substantially exceeds the observed NF-κB range, the Hill function operates in the low saturation regime, where its series approximation up to first order around z=0 gives $\hat{U}(z)\approx 1.668+1.148\cdot z^{2.114}$. This close agreement between two independent fitting procedures provides evidence that the NF-κB-mediated regulation of p53 synthesis follows Hill-type kinetics with a coefficient n≈2, indicating cooperative binding. For the Hunziker-UDE, the weak magnitude of the crosstalk effect prevents analogous conclusions. Nevertheless, both the symbolic regression and Hill-type regression yield a monotonically increasing function, reinforcing the recent experimental finding that NF-κB positively modulates p53 synthesis (Colombo *et al*. 2026).

## 4 Discussion

In this work, we demonstrate that universal differential equations can be applied to determine unknown crosstalk functions between signaling pathways from real experimental single-cell data exhibiting complex oscillatory dynamics. We first validate the framework on synthetic benchmarks, showing that it reliably recovers ground-truth crosstalk functions of varying shape and strength, with recovery quality improving markedly with the number of available time series and remaining robust to measurement noise across the tested range. We then apply the framework to 106 simultaneously measured NF-κB and p53 time series, embedding a NN into mechanistic p53 ODE models to identify a monotonically increasing crosstalk function in which elevated NF-κB levels enhance p53 synthesis. Symbolic regression distills the learned neural network output into a compact closed-form expression, providing the first quantitative, time-resolved characterization of NF-κB-driven amplification of p53 response to irradiation at the single-cell level that was recently described experimentally (Colombo *et al*. 2026).

Both the Konrath-based and Hunziker-based UDE models consistently identify a crosstalk function that increases with NF-κB, lending further confidence to the finding that it positively modulates p53 production. These results provide a methodologically orthogonal confirmation to the data described in Colombo *et al*. (2026), by which there is an NF-κB mediated transcriptional increase of *TP53*. Of note, this finding goes against the common view of both transcription factors as functionally antagonistic; instead, our work provides additional evidence pointing to a possible “molecular amplification” of the p53 response through NF-κB activation. Altogether, it shows how UDE-based data analysis and fitting pipelines can contribute to confirm and consolidate experimental observations. Furthermore, it might also provide guidance for new experimental explorations. Specifically, we identify a Hill function with a certain degree of cooperativity as plausible coupling between the dynamics of both transcription factors. A genetic perturbation of the *TP53* promoter that adds or removes binding sites should change the resulting p53 dynamics upon co-activation with NF-κB which could be assessed experimentally. We cannot exclude that in certain contexts a mutation causes an analogous effect, hence altering the “physiological” crosstalk between the two signaling pathways. Similarly, epigenetic regulation of gene expression might differ between cells and influence this crosstalk. These scenarios would be worthwhile to characterize further, both biologically and computationally with our developed methods.

The two mechanistic p53 models differ substantially in the inferred strength of the crosstalk effect, with the Konrath-based UDE indicating stronger effects of NF-κB. We consider the Konrath-based result more biologically plausible: The model provides a more detailed mechanistic representation of p53 regulation, explicitly including the known feedback regulators Mdm2 and Wip1 as well as the upstream kinase ATM than the simpler Hunziker model which lacks these components. Accordingly, the Konrath-UDE achieves a better fit to the single-cell trajectories. In the Hunziker-UDE, the reduction in degrees of freedom may cause the neural network to partially compensate for missing regulatory interactions rather than exclusively capturing the NF-κB crosstalk.

Our framework assumes that NF-κB modulates p53 dynamics exclusively through its synthesis rate, which we consider the most parsimonious assumption given the current experimental evidence (Colombo *et al*. 2026). However, we cannot distinguish whether this effect acts at the level of transcription, mRNA stability, or translation, although since NF-κB is a transcription activator, the first option is the most likely. Further, the UDE approach suffers from the same limitations as classical systems biology approaches: proving or disproving qualitatively (and thus also quantitatively) different hypotheses on mechanisms of regulation remains difficult. More complex crosstalk architectures where the NF-κB signal affects the p53 pathway at multiple points have been suggested (Konrath *et al.* 2020). In that study, due to lack of temporal resolution of the NF-κB signal, crosstalk was restricted to NF-κB-level independent, global relative changes in kinetic parameters. As we consider NF-κB activation levels to directly translate to altered influence on p53, our approach provides complementary and not contradicting insights under a different set of assumptions.

Previous applications of UDEs to real biological data involved systems with smooth, simple dynamics and few time points. The p53 system poses a more complex inference problem. Each cell exhibits multiple oscillatory pulses, both in p53 and NF-κB, with distinct amplitudes, frequencies, and damping. What further distinguishes our approach is the availability of data from 106 independent cells, each oscillating differently, that a single neural network as common crosstalk function must simultaneously explain. This strongly constrains the optimization to converge on the shared crosstalk function across the cell population. Our synthetic experiments confirm this: recovery quality improves dramatically between 16 and 128 time series, and sample size emerges as the dominant factor over noise level. It is this combination of dynamically complex data, a large number of independent biological replicates, and a shared neural network architecture that moves the inference beyond parameter estimation toward genuine mechanism recovery, and suggests that UDEs are ready to be applied to the increasingly complex datasets that modern single-cell measurement technologies produce. At the same time, while central to identifying a common mechanism, our assumption on a single crosstalk function shared across all cells may mask genuine cell-to-cell differences in how NF-κB modulates p53 synthesis and hampers fitting cell-specific crosstalk. Conditional UDE architectures (de Rooij *et al.* 2025b) could accommodate such heterogeneity, though at the cost of an increased risk of overfitting. Scaling to more complex processes such as cancer and inflammation is an exciting future direction. It requires richer datasets to constrain the larger parameter space, a challenge inherent to all mechanistic modeling approaches.

As a limitation, we do not model potential time delays between NF-κB activation and its effect on p53, which would require delay differential equations and substantially increase numerical complexity. Additionally, the optimized initial conditions do not necessarily coincide with the unstimulated steady states, and the synthetic noise model does not capture systematic discrepancies between the ODE structure and the true biology, both of which may affect the learned crosstalk function. Despite these limitations, our results establish UDE-based scientific discovery as a viable approach for learning unknown interactions in biological signaling networks from real experimental data, providing a principled pipeline from noisy single-cell measurements to interpretable mathematical descriptions of pathway crosstalk.

## Supplementary Material

btag449_Supplementary_Data

## Data Availability

The data underlying this work were generated by Colombo *et al.* (2026) and are available in the Supplementary Material of that work, at https://doi.org/10.64898/2026.02.24.707448. No new data were generated in support of this research.
