# Supplementary material for: Universal differential equations for quantifying NF-κB—p53 signaling crosstalk
Source: Bioinformatics. 2026 Aug 21;42(Suppl 2):btag449. doi: 10.1093/bioinformatics/btag449 (PMC13501283; doi:10.1093/bioinformatics/btag449)
Supplement: Supplementary file 1 [file btag449_supplementary_data.pdf]

Supplementary Material

Universal differential equations for quantifying NF-κB - p53 signaling crosstalk

Umur Can Kaya[1,2], Xhemal Kodragjini[1], Samuel Zambrano[3,4] and Katharina Baum[1,2*]

[1]Department of Mathematics and Computer Science, Freie Universität Berlin, 14195 Berlin, Germany
[2]Hasso-Plattner-Institute, Digital Engineering Faculty, University of Potsdam, 14482 Brandenburg, Germany
[3]Università Vita-Salute San Raffaele, 20132 Milan, Italy
[4]Division of Genetics and Cell Biology, IRCCS Ospedale San Raffaele, 20132 Milan, Italy
[*]Corresponding author: katharina.baum@fu-berlin.de

Contents

1 Experimental p53 and NF-κB time series data **2**

2 Hunziker Model **2**

3 Konrath Model **3**

4 Implementation details **5**

5 Neural network architecture **5**

6 Optimization details **5**

7 Selection of diverging ensemble members **6**

8 Cross-validation of the crosstalk function **7**

9 Symbolic regression for quantifying the crosstalk function **8**

10 Ground-truth crosstalk functions for synthetic data benchmark **9**

11 Synthetic data generation **9**

12 Evaluation metrics **10**

13 Synthetic experiment results **11**

14 Kinetic parameter distributions **12**

15 Hill regression & series approximation for quantifying the crosstalk function **13**
15.1 Derivation of the series approximation 13

1 Experimental p53 and NF-κB time series data

Data was obtained from live cell imaging experiments performed in (Colombo et al., 2026). Therein, images of MCF-7 cells expressing fluorescently tagged versions of p53 and NF-κB were recorded every 6 min for 20 h using an adapted microscopy system. For the dataset hereby considered, two stimuli activating p53 and NF-κB were used: 10 Gy gamma irradiation and 10 ng/ml TNF-α, respectively. It consists of paired time series (p53 and NF-κB) of 106 cells in total. The following imaging analysis pipeline was used: before the experiment, the cells were stained with Hoechst 33342, a nuclear dye which allows subsequent nuclear segmentation and tracking. Specifically, the AI-based segmentation system Cellpose (Stringer et al., 2020) was used, which allows to segment the nuclear and cytosolic cell fractions; cells were then tracked using the Hungarian linker as described previously (Careccia et al., 2019). In particular, we rely on data from the Supplementary Material - Raw Data as data provided in relation to Fig. 3 (`https://www.biorxiv.org/content/biorxiv/early/2026/02/25/2026.02.24.707448/DC2/embed/media-2.zip?download=true`) from (Colombo et al., 2026). Following other studies, the p53 time series correspond to background-corrected mean intensity nuclear levels of the fluorescently tagged p53, whereas NF-κB time series correspond to the nuclear to cytosolic ratio of the fluorescence average intensity, a usual proxy for NF-κB nuclear concentration.

2 Hunziker Model

This p53 signaling ODE model stems from Hunziker et al. (2010).

$$\frac{dp}{dt} = \sigma_p \cdot c_f - \alpha \cdot p - k_f \cdot p \cdot m + k_b \cdot c + \gamma \cdot c \quad (1)$$

$$\frac{dm_m}{dt} = k_t \cdot p^2 - \beta \cdot m_m \quad (2)$$

$$\frac{dm}{dt} = k_{tl} \cdot m_m - k_f \cdot p \cdot m + k_b \cdot c + \delta \cdot c - \gamma \cdot m \quad (3)$$

$$\frac{dc}{dt} = k_f \cdot p \cdot m - k_b \cdot c - \delta \cdot c - \gamma \cdot c \quad (4)$$

where p denotes nuclear p53 protein, m_m Mdm2 mRNA, m Mdm2 protein, and c the p53–Mdm2 complex. k_f and k_b are the complex formation and dissociation rate constants, respectively, γ the Mdm2 degradation rate, and c_f is the crosstalk factor.

Table S1: Nominal kinetic parameter values for the Hunziker model at initialization.

Parameter	Symbol	Value
`sigma_p`	σ_p	720.5
`alpha`	α	0.018
`gamma`	γ	0.042
`k_b`	k_b	7536
`r`	r	1.134
`k_trs`	k_t	0.008
`beta`	β	0.428
`k_trd`	k_{tl}	0.2
`delta`	δ	0.9
`a`	a	21.240
`b`	b	2.0

3 Konrath Model

This p53 signaling ODE model stems from Konrath et al. (2020).

$$\frac{d(p53)}{dt} = \beta_p \cdot c_f + \alpha_{wpa} \cdot Wip1 \cdot p53a - \alpha_{pi} \cdot p53 - \alpha_{mpi} \cdot Mdm2 \cdot p53 - \beta_{sp} \cdot p53 \cdot \frac{ATMp^{ns}}{ATMp^{ns} + T_s^{ns}} \tag{5}$$

$$\frac{d(p53a)}{dt} = \beta_{sp} \cdot p53 \cdot \frac{ATMp^{ns}}{ATMp^{ns} + T_s^{ns}} - \alpha_{mpa} \cdot Mdm2 \cdot p53a - \alpha_{wpa} \cdot Wip1 \cdot p53a \tag{6}$$

$$\frac{d(\text{Mdm2 mRNA})}{dt} = \beta_{mt} + \beta_{pamt} \cdot p53a - \alpha_{mt} \cdot \text{Mdm2 mRNA} \tag{7}$$

$$\frac{d(\text{Mdm2})}{dt} = \beta_{mtm} \cdot \text{Mdm2 mRNA} - \alpha_{sm} \cdot Mdm2 \cdot ATMp - \alpha_m \cdot Mdm2 \tag{8}$$

$$\frac{d(\text{Wip1 mRNA})}{dt} = \beta_{wt} + \beta_{pawt} \cdot p53a - \alpha_{wt} \cdot \text{Wip1 mRNA} \tag{9}$$

$$\frac{d(\text{Wip1})}{dt} = \beta_{wtw} \cdot \text{Wip1 mRNA} - \alpha_w \cdot \text{Wip1} \tag{10}$$

$$\frac{d(ATMp)}{dt} = \beta_s \cdot \texttt{damage} - \alpha_{ws} \cdot ATMp \cdot \frac{Wip1^{nw}}{Wip1^{nw} + T_w^{nw}} - \alpha_s \cdot ATMp \tag{11}$$

Where c_f is the crosstalk factor.

Table S2: Nominal kinetic parameter values for the Konrath model at initialization.

Parameter	Symbol	Value
bp	β_p	2.307
api	α_{pi}	10^{-10}
ampi	α_{mpi}	0.00544
awpa	α_{wpa}	2.418
bsp	β_{sp}	9999.58
ampa	α_{mpa}	0.0802
bmt	β_{mt}	20.773
bpamt	β_{pamt}	96.459
amt	α_{mt}	0.520
bmtm	β_{mtm}	1.592
asm	α_{sm}	14.662
am	α_m	1.0
bwt	β_{wt}	10^{-10}
bpawt	β_{pawt}	13.428
awt	α_{wt}	1.0
bwtw	β_{wtw}	0.110
aw	α_w	1.0
bs	β_s	1.912
aws	α_{ws}	9998.04
as	α_s	10^{-10}
Ts	T_s	30.640
Tw	T_w	13.255
ns	n_s	4.0
nw	n_w	4.0
a	a	21.240
b	b	149.116

4 Implementation details

We implement the UDE models using the Python JAX library (Bradbury et al., 2018), which enables efficient automatic differentiation and just-in-time compilation. We solve the differential equations using the Diffrax library (Kidger, 2021). Due to the stiffness of the biological systems, we employ the `Kvaerno5` (Kværnø, 2004) solver, a 5th-order implicit Runge-Kutta method. The integration step size is adapted dynamically using a PID controller (Söderlind, 2003) to maintain a relative tolerance of 10^{-2} and an absolute tolerance of 10^{-4}. Gradients are computed via the `RecursiveCheckpointAdjoint` method (Stumm and Walther, 2010; Wang et al., 2009), which is memory-efficient for stiff systems. To ensure stable integration, we set the initial step size (dt_0) based on the model complexity: $dt_0 = 0.1$ for the Hunziker model and $dt_0 = 0.01$ for the Konrath model. We implement the neural networks using the Equinox library (Kidger and Garcia, 2021). We use the AdamW (Loshchilov and Hutter, 2019) optimizer from the Optax library (DeepMind et al., 2020) with a learning rate of 10^{-3} and weight decay of 10^{-5} to regularize the neural network weights. To ensure numerical stability during backpropagation through the ODE solver, we employ gradient clipping with a global norm of 1. For the synthetic data benchmarks, we use a mini-batch size of $B = 16$ and train for 1000 epochs. For the real data experiments, we set $B = 41$ and train the model for 3000 epochs. Data is shuffled at the start of each epoch to ensure diverse mini-batches.

5 Neural network architecture

The neural network receives the scalar NF-κB signal $z(t)$, obtained via linear interpolation of the measured time series, and outputs a scaling factor that modifies the basal p53 synthesis rate s_{p53} (parametrized by σ_p in Hunziker, β_p in Konrath models) to obtain the effective p53 production rate $s_{p53-\text{eff}}$. The architecture of the neural network is a multi-layer perceptron consisting of a single input neuron, two hidden layers with 16 neurons each, and a single output neuron. We use the rectified linear unit (ReLU) (Nair and Hinton, 2010) activation function for the hidden layers, and a softplus activation function in the output layer to ensure biologically meaningful non-negative synthesis rates.

6 Optimization details

The kinetic parameters $\mathbf{p}_m$ and initial conditions $\mathbf{x}_{0,m}$ must remain within biologically plausible, strictly positive ranges throughout training. To enforce this without modifying the optimizer, we employ a sigmoid reparametrization: each constrained parameter $p \in [p_{\min}, p_{\max}]$ is represented internally as an unconstrained real-valued variable $\tilde{p} \in \mathbb{R}$, and transformed back via:

$$p = p_{\min} + (p_{\max} - p_{\min}) \cdot \frac{1}{1 + e^{-\tilde{p}}},$$

The optimizer operates entirely on $\tilde{p}$, while the ODE solver always receives the physically valid p. The bounds are set to $p_{\min} = 0.1 \cdot \min_m p_{m,0}$ and $p_{\max} = 1.9 \cdot \max_m p_{m,0}$, where $p_{m,0}$ denotes the initialization of parameter p for cell m, restricting the search to a biologically plausible neighborhood of the published nominal values and preventing the UDE dynamics from diverging during training. The same scheme is applied to the initial conditions. To prevent numerical instability and solution divergence in the stiff regime UDE integration, the state variables are additionally clipped to $[0, 10^6]$ at each evaluation of the right-hand side of the UDE. The measurement parameters a_m and b_m, having no strict positivity requirement within the loss landscape, are optimized directly without reparameterization.

The optimization of the full parameter set requires careful initialization, as the non-convex nature of the UDE training landscape makes the final solution sensitive to the choice of initialization (Schmid et al., 2025). The kinetic ODE parameters for each cell m, $\mathbf{p}_m$, are initialized from published nominal values reported by (Hunziker et al., 2010; Konrath et al., 2020). Since each cell receives its own independent parameter vector, the M per-cell initializations are generated by sampling from a log-normal distribution with mean p and standard deviation $0.25 \cdot p$ for each nominal parameter value p. This preserves strict positivity of all kinetic parameters and, together with the random initialization of the neural network weights, provides diverse initializations for the independent ensemble runs described in Section 2.3.3 of the main text.

Prior to UDE training, the initializations (for optimization) of the initial conditions $\mathbf{x}_{0,m}$ (for ODE integration) are determined through a pre-equilibration phase of the respective unstimulated ODE systems without DNA damage. The respective ODE model is first integrated over a pre-equilibration period of approximately 72 hours. The final states of these trajectories are then used as initializations of the initial conditions for the subsequent UDE training on the stimulated system. Note that the initial conditions are subsequently optimized independently from the kinetic parameters of the ODE system, i.e., final fitted initial condition values for fitting stimulated p53 time series are not coinciding with unstimulated steady states.

Finally, the measurement parameters a_m and b_m are initialized to global constants shared across all cells at the start of training (but then optimized separately for each cell). For the Konrath model, these constants are taken from (Konrath et al., 2020), whereas for the Hunziker model, the background offset is the same as for the Konrath model, and the scaling factor is chosen based on the relation between amplitudes in the published model and the observed data.

7 Selection of diverging ensemble members

Each ensemble consists of $N_{\text{ens}} = 10$ independent training runs, each initialized with a different random seed that diversifies the neural network weights, the per-cell kinetic parameter samples, and the mini-batch ordering. As is common in high-dimensional nonlinear parameter estimation (Westny et al., 2024; Gutenkunst et al., 2007), a minority of ensemble members encounter numerical instabilities during optimization. These manifest as `NaN` (not a number) values in the training loss, arising when the ODE solver produces non-finite state trajectories for a particular combination of initial parameters and mini-batch ordering. Diverging runs are detected automatically at the end of training by checking whether the final loss value is finite. All runs that produce a finite final loss are retained, yielding a final ensemble size of $N_{\text{ens}} \geq 8$ in all reported experiments. The crosstalk function estimate $U_\theta(z)$ and all reported error metrics are computed exclusively from the retained ensemble members.

8 Cross-validation of the crosstalk function

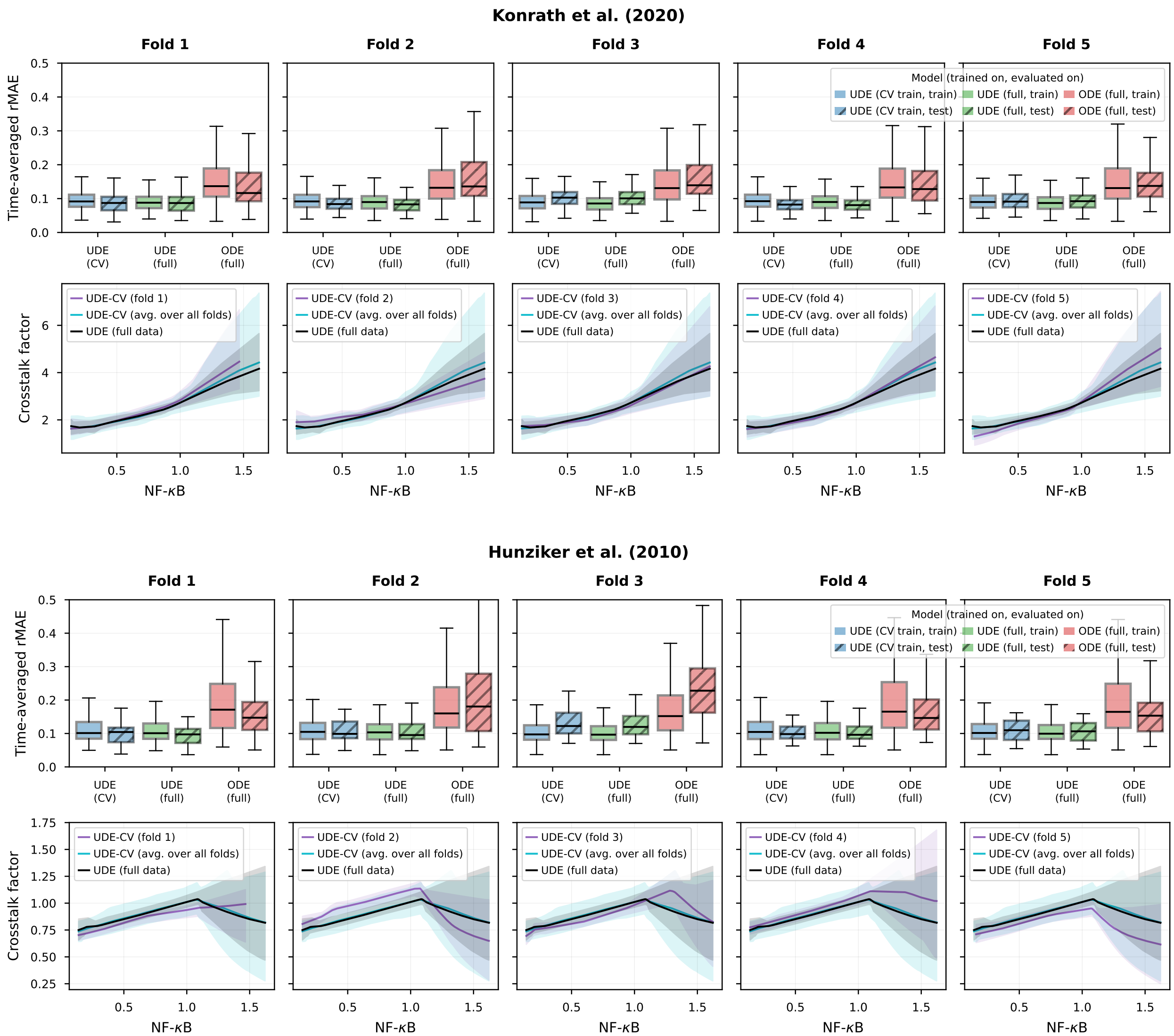

Figure S1: 5-fold cross-validation results for the Konrath (top) and Hunziker (bottom) UDE models. Each column corresponds to one fold. (Upper rows) Boxplots of the time-averaged rMAE of the measured p53 time series across cells for three models: the CV UDE (trained on 84 or 85 cells, with the NN frozen for evaluation on held-out cells), the full-data UDE (trained on all 106 cells), and the full-data ODE baseline. Filled boxes show performance on training cells; hatched boxes show performance on the held-out test cells. For both models and all folds, the CV UDE test rMAE (dark hatched boxes) matches the training rMAE, and both are substantially lower than the ODE baseline, confirming that the learned crosstalk function supports fitting the observed p53 time series better also in unseen cells. (Lower rows) Crosstalk function $U_\theta(z)$ recovered from each fold's training cells (purple), the average across all five folds (cyan), and the full-data UDE fit (black); shaded bands show the 5th–95th ensemble percentile. The per-fold functions are mutually consistent and closely reproduce the full-data fit, demonstrating that the inferred crosstalk function shape is stable across different subsets of cells and thus probably not an artifact of overfitting.

The UDE parameter set $\Phi = \{\theta\} \cup \{\mathbf{p}_m, \mathbf{x}_{0,m}, a_m, b_m\}_{m=1}^{M}$ consists of two structurally distinct components. The per-cell parameters $\{\mathbf{p}_m, \mathbf{x}_{0,m}, a_m, b_m\}$ capture cell-to-cell variability in p53 signaling dynamics and are estimated independently for each cell. The neural network weights θ, by contrast, encode the uni-

versal crosstalk function $U_\theta(z)$ shared across all $M = 106$ cells; they are the only component that could in principle overfit to the particular cells used during training.

To assess whether U_θ generalizes to cells not seen during training, we perform a 5-fold cross-validation. The $M = 106$ cells are randomly permuted using a fixed seed and partitioned into five folds as evenly as possible: one fold contains 22 test cells and the remaining four contain 21, yielding training sets of 84 or 85 cells, respectively. For each fold $f \in \{1, \ldots, 5\}$, two sequential optimization phases are carried out. In the training phase, the full UDE, neural network weights θ together with all per-cell parameters for the $M_{\text{train}} \in \{84, 85\}$ training cells, is trained jointly on the training cells using the identical procedure described in Sections 4 and 6, including pre-equilibration, sigmoid reparametrization, ensemble initialization with $N_{\text{ens}} = 10$ independent runs, and $N_{\text{epochs}} = 3000$ epochs of AdamW optimization. In the test phase, the trained neural network weights $\hat{\theta}^{(f)}$ are frozen; only fresh per-cell parameters $\{\mathbf{p}_m, \mathbf{x}_{0,m}, a_m, b_m\}$ are optimized for the $M_{\text{test}} \in \{21, 22\}$ held-out test cells, again following the standard training procedure with $\hat{\theta}^{(f)}$ providing a fixed, non-trainable crosstalk function. We evaluate generalization by comparing the reconstruction of the p53 time series using time-averaged rMAE (Section 12) on held-out test cells, fitted with the frozen neural network weights, against the rMAE on training cells. A substantially higher test rMAE for the p53 measurements would indicate that U_θ has overfit to the specific oscillatory patterns of the training cells rather than capturing a universal crosstalk mechanism. Figure S1 (upper row) shows that this is not the case: across all five folds and for both the Konrath and Hunziker models, the distribution of per-cell test rMAE on p53 is indistinguishable from the training rMAE distribution, and both remain substantially below the error of the p53 reconstruction for the ODE baseline (without any crosstalk function) evaluated on the same cells. The ODE model, which contains no neural network component and is therefore immune to the scrutinized overfitting by construction, serves as a reference confirming that the improvement of the UDE over the ODE is a genuine signal. Furthermore, the rMAE of the full-data UDE, trained on all 106 cells simultaneously, closely matches both the CV training and CV test rMAE, demonstrating that using the full dataset introduces neither additional bias nor overfitting. Training on all available cells is therefore strictly preferable, as also indicated by our synthetic experiment, as it maximally constrains the shared neural network and provides more correct solutions for the inferred crosstalk function.

Figure S1 (lower row) shows the crosstalk function $U_\theta(z)$ recovered from each fold's training cells alongside the full-data fit. The per-fold crosstalk functions are mutually consistent across the entire observed NF-κB range and agree closely with the full-data estimate.

9 Symbolic regression for quantifying the crosstalk function

While direct sparse regression methods like the Sparse Identification of Nonlinear Dynamics (SINDy) (Brunton et al., 2016) and its variants are highly effective for discovering identifiable biological models, we employ a two-step pipeline: first optimizing the UDE with a neural network, then applying symbolic regression to the learned function. We chose this approach over SINDy-style methods because the latter requires committing to a basis library of candidate functions before fitting. As highlighted by Massonis et al. (2023), formulating this candidate library is a crucial dependency of such algorithms, and the need to capture complex or high degree terms can substantially complicate the model discovery process. This choice affects not only the final symbolic regression result but the ODE fit itself: if the true crosstalk function is not well represented by the chosen library, the ODE is forced to compensate through other parameters, potentially distorting kinetic parameter estimates and degrading the fit. Because the functional form of the NF-κB-p53 crosstalk is precisely what we aim to discover, specifying a library upfront (and potentially affecting the fit of the other parameters) would mean confirming a hypothesis rather than discovering a relationship. The neural network makes no such assumption; as a universal approximator, it places no restriction on the crosstalk shape, leaving the ODE fit unconstrained by any symbolic prior. Symbolic regression is then applied post hoc to the frozen, data-driven neural network predictions, ensuring that the kinetic parameter estimation remains entirely independent of the chosen symbolic function library.

To obtain an interpretable closed-form approximation of the discovered crosstalk function, we apply symbolic regression post hoc to the ensemble mean of the learned neural network output $U_\theta(z)$. We use the PySR library (Cranmer, 2023), which searches the space of mathematical expressions using a multi-population evolutionary algorithm and has recently been shown to outperform competing symbolic regression

approaches on a range of dynamical systems (Brum et al., 2026).

After all ensemble training runs are complete, $U_\theta(z)$ is evaluated for each run on the NF-κB values observed in that run. The ensemble mean and standard deviation are then computed on a uniform grid of 1000 points spanning the intersection of the NF-κB ranges across all runs. To account for varying reliability of the ensemble estimate across the NF-κB range, each grid point is weighted by the inverse variance of the ensemble predictions, $w_i = 1/\hat{s}_i^2$, so that regions of high ensemble agreement contribute more to the fit.

The PySR search uses the binary operators $\{+, -, \times, \div, \text{pow}\}$ with no unary operators. Operator complexities are set to 1 for $+, -, \times, \div$ and 3 for pow. A parsimony penalty of 1 and a maximum expression size of 7 nodes are applied to strongly favor compact expressions and prevent overfitting with high-degree polynomials. The algorithm runs for 50 iterations and selects the best equation by PySR score. The search is made fully deterministic by fixing the random seed and running in serial mode.

Applying this symbolic regression to the ensemble mean crosstalk functions yields the following quadratic function in NF-κB z for the Konrath-based model:

$$\hat{U}_{\text{Konrath}}(z) = z^2 + 1.698.$$

For the Hunziker-based model, we obtain a linear function:

$$\hat{U}_{\text{Hunziker}}(z) = 0.274 \cdot z + 0.717.$$

10 Ground-truth crosstalk functions for synthetic data benchmark

For creating our synthetic benchmark datasets, we define four distinct ground-truth crosstalk functions $g(z)$, depending on NF-κB at time t, $z(t)$, based on either weakly or strongly activating (increasing) or inhibitory (decreasing) Hill-type kinetics. They are defined as:

$$g_{\text{act}}(z) = c_{\text{off}} + c_{\text{sc}} \frac{z^n}{K_d^n + z^n}, \text{ or}$$
$$g_{\text{inh}}(z) = c_{\text{off}} + c_{\text{sc}} \frac{K_d^n}{K_d^n + z^n}.$$

For all functions, the dissociation constant is set to $K_d = 0.8$, the Hill coefficient to $n = 3.0$, and the basal offset to $c_{\text{off}} = 0.8$. To simulate weak crosstalk, the scaling factor is set to $c_{\text{sc}} = 1.5$, while strong crosstalk is modeled using $c_{\text{sc}} = 12.0$.

For each crosstalk scenario, the ground-truth crosstalk function enters the p53 ODE models of Konrath or Hunziker via altering the p53 synthesis s_{p53} rate (parametrized by σ_p in the Hunziker model and β_p in the Konrath model) such that the effective p53 synthesis rate becomes

$$s_{p53-\text{eff}}(z(t)) = s_{p53} \cdot g(z)$$

for an NF-κB signal $z(t)$ at time t.

11 Synthetic data generation

Synthetic NF-κB time series are generated by sampling with replacement from the measured NF-κB dataset (Section 2.1 of the main text). To introduce realistic cell-to-cell variability while strictly maintaining positive concentration values, each sampled time series is scaled by a multiplicative factor drawn from a log-normal distribution with a median of 1.0 and a standard deviation of 0.05.

Using these synthetic NF-κB signals and one of the four ground-truth crosstalk functions defined in Section 10, synthetic p53 time series are generated by simulating trajectories using both the Hunziker and the Konrath ODE models, retaining one time point every 6 min up to 20h.

To systematically assess the data requirements and noise robustness of the UDE framework, the number of available single-cell time series M is varied across three levels: 16, 128, and 512 samples.

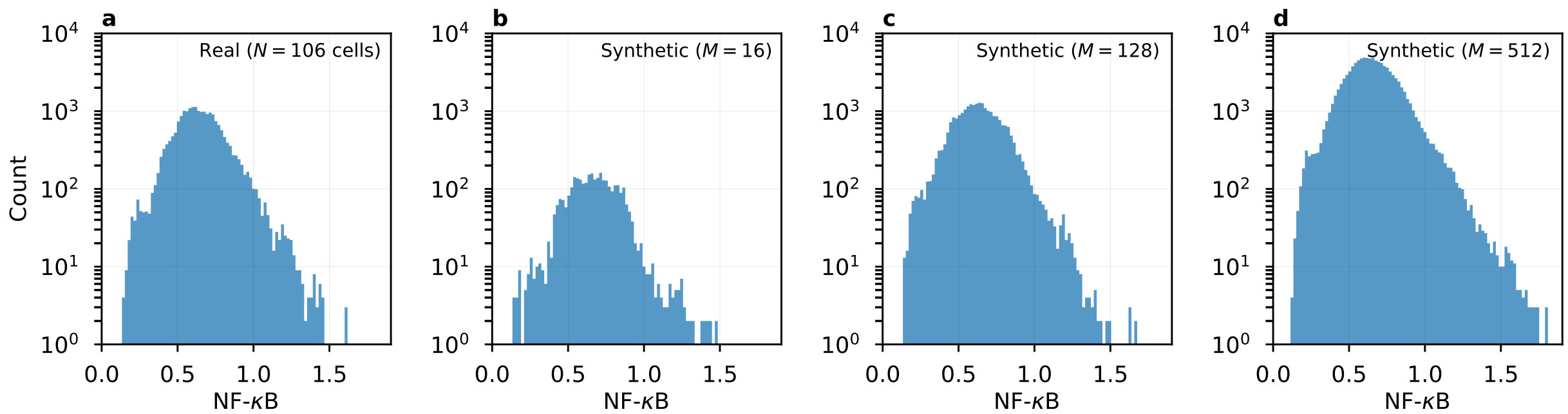

Figure S2: NF-κB value distributions for real and synthetic datasets. Histograms of all NF-κB measurements pooled across time points and cells (log-scale count). Panel (a) shows the full real dataset ($N = 106$ cells). Panels (b–d) show synthetic datasets generated by sampling with replacement at $M = 16$, 128, and 512 cells, respectively, each with log-normal multiplicative scaling.

To mimic experimental measurement artifacts, multiplicative log-normal noise is injected into the generated p53 time series, where each time point is scaled by $\exp(\varepsilon)$ with $\varepsilon \sim \mathcal{N}(0, \eta^2)$, at three noise levels $\eta \in \{0.001, 0.01, 0.1\}$.

Synthetic p53 time series are generated using the same ODE solver settings and NF-κB interpolation precedure as the UDE training pipeline. Model parameters for each synthetic cell are drawn from a log-normal distribution centered on the published nominal values with a coefficient of variation of 10%, compared to 25% used for UDE training initialization.

12 Evaluation metrics

To assess the quality of the model fit to the M observed p53 time series and NF-κB input signals, we compute the relative mean absolute error (rMAE) per time point:

$$\mathrm{rMAE}(t_k) = \frac{\frac{1}{M}\sum_{m=1}^{M} |y_{m,k} - \hat{y}_m(t_k, z_k)|}{\frac{1}{M}\sum_{m=1}^{M} y_{m,k}},$$

where $y_{m,k}$ is the measured p53 signal of cell m at time t_k and $\hat{y}_m(t_k, z_k)$ is the corresponding model prediction. Normalizing by the population mean at each time point makes the error comparable across the dynamic range of the signal. When reporting mean rMAE, the mean is calculated across the ensemble members and time points.

For the synthetic data experiments, where the ground-truth crosstalk function $g(z)$ is known, we quantify crosstalk recovery using the relative mean absolute error (rMAE):

$$\mathrm{rMAE} = \frac{\frac{1}{G}\sum_{i=1}^{G} |U_\theta(z_i) - g(z_i)|}{\frac{1}{G}\sum_{i=1}^{G} |g(z_i)|},$$

where $\{z_i\}_{i=1}^{G}$ is a uniform grid of ($G = 500$) NF-κB values spanning the range observed in the data and $U_\theta(z_i)$ is the prediction of the learned crosstalk function.

13 Synthetic experiment results

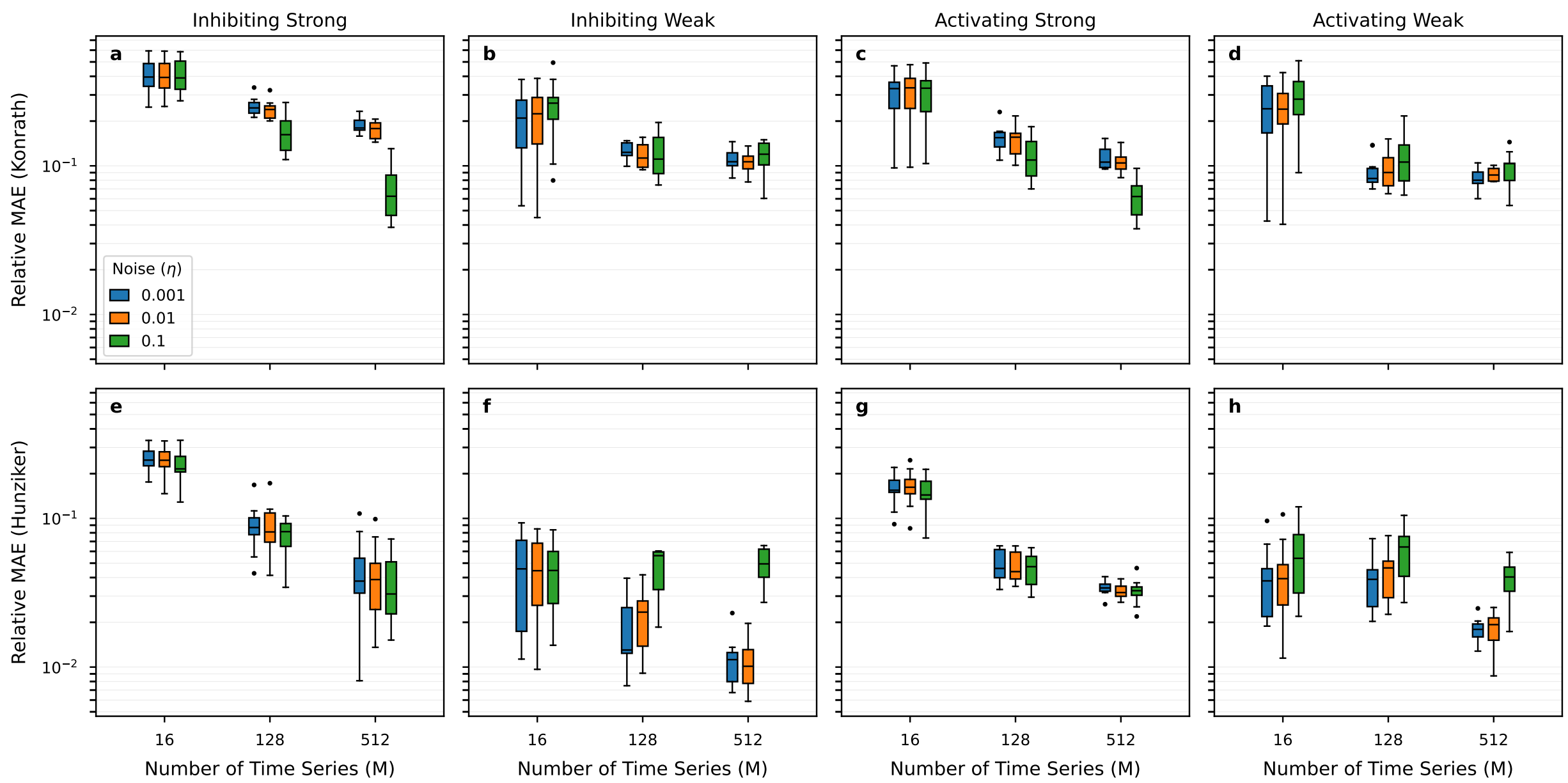

Figure S3: Relative MAE of crosstalk function recovery across all synthetic benchmark conditions. Each panel shows boxplots of the relative MAE between the learned crosstalk function $U_\theta(z)$ and the ground-truth $g(z)$, evaluated over 10 independent ensemble runs. Columns correspond to the four ground-truth crosstalk scenarios (Inhibiting Strong, Inhibiting Weak, Activating Strong, Activating Weak); rows correspond to the Konrath (a–d) and Hunziker (e–h) ODE models. Within each panel, results are grouped by number of single-cell time series $M \in \{16, 128, 512\}$ and colored by noise level $\eta \in \{0.001, 0.01, 0.1\}$.

14 Kinetic parameter distributions

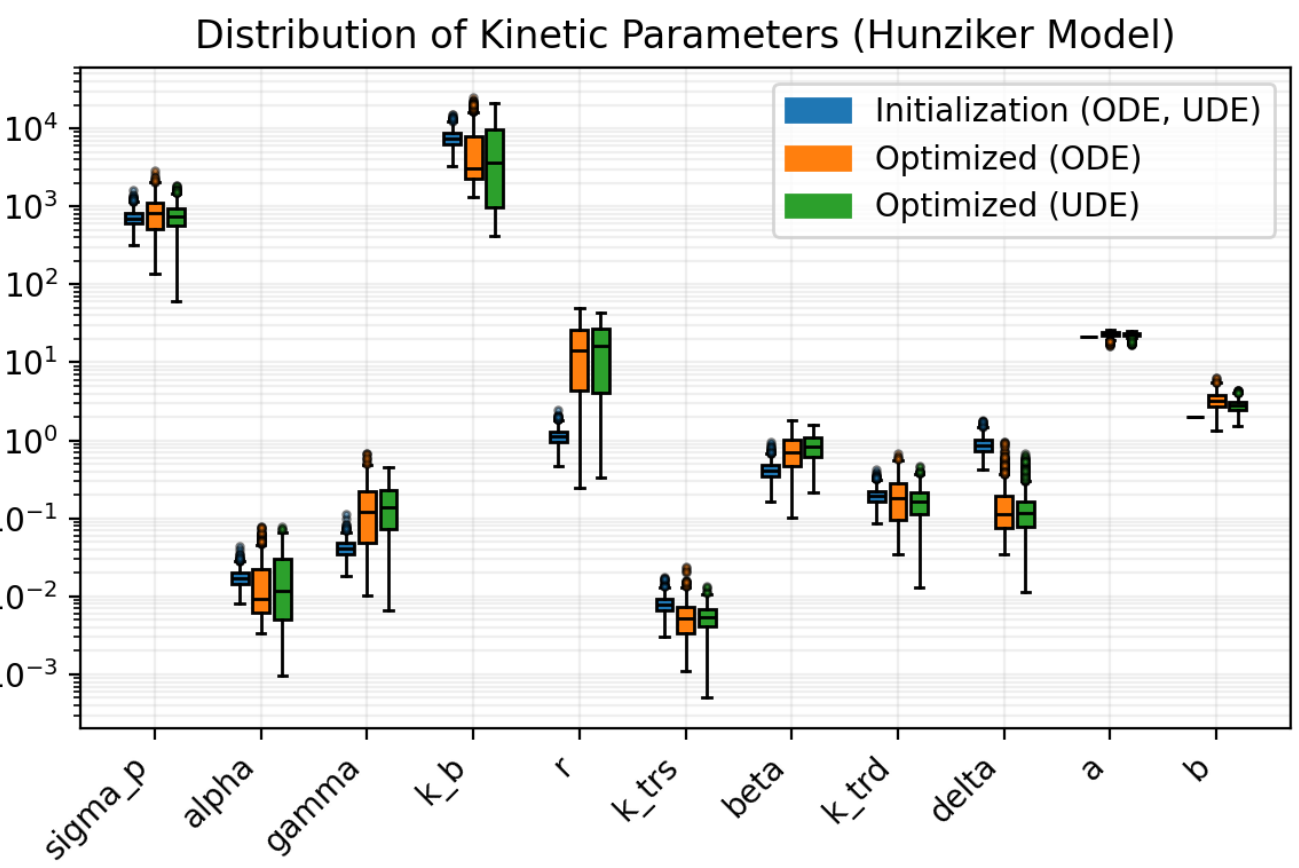

Figure S4: Per-cell kinetic parameter distributions for the Hunziker model. Boxplots of each kinetic parameter and measurement parameter (a, b) across all cells, shown on a log scale. Blue: initialization values (shared between ODE and UDE); orange: values optimized by the ODE-only model; green: values optimized by the UDE model.

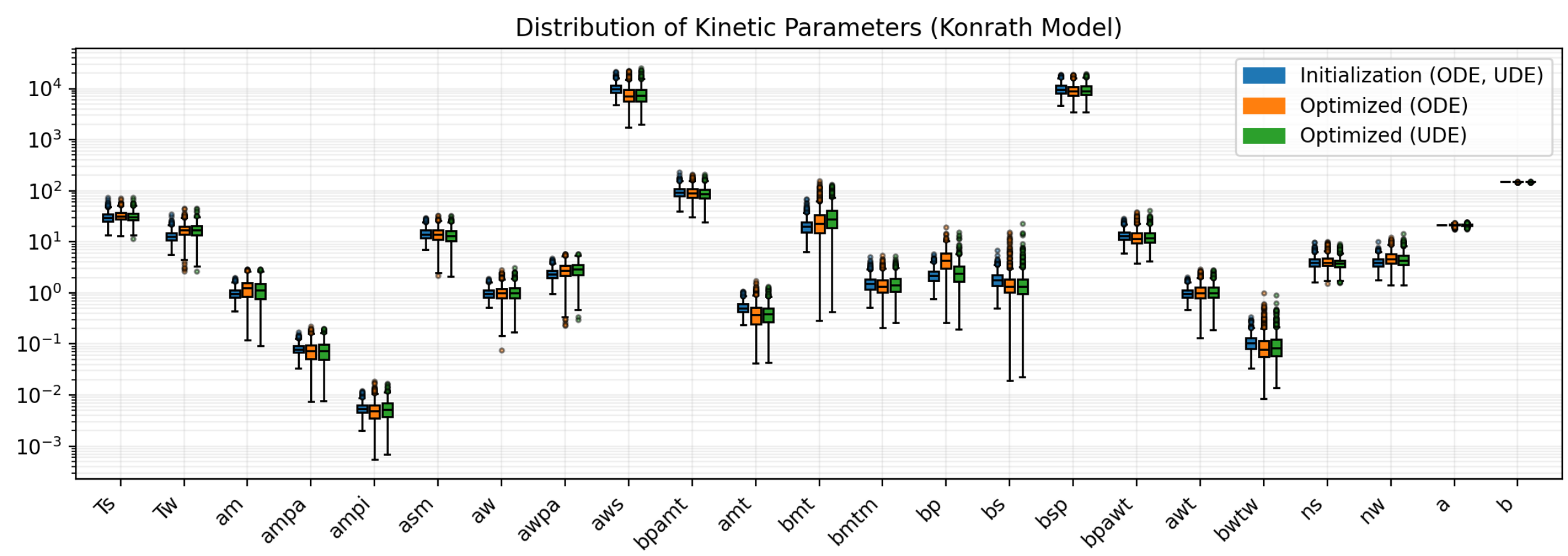

Figure S5: Per-cell kinetic parameter distributions for the Konrath model. Boxplots of all kinetic and measurement parameters (a, b) across cells on a log scale. Blue: initialization; orange: ODE-optimized; green: UDE-optimized. Parameters α_{pi}, α_s, and β_{wt} are excluded as they were set to 10^{-10} in the original Konrath model (Konrath et al., 2020) and remained at this value throughout optimization. `damage` is omitted as it is a fixed external input rather than a trainable parameter.

15 Hill regression & series approximation for quantifying the crosstalk function

As a complementary, interpretable alternative to symbolic regression, we fit constrained Hill-type functions directly to the ensemble mean of the learned neural network crosstalk output $U_\theta(z)$. After all ensemble training runs are complete, $U_\theta(z)$ is evaluated for each run on the NF-κB values observed in that run. The individual run predictions are then interpolated onto a common uniform grid of 1000 points spanning the NF-κB range, and the ensemble mean $\bar{U}(z)$ and standard deviation $\hat{s}(z)$ are computed at each grid point.

Two candidate Hill functions are fitted to $\bar{U}(z)$ using nonlinear least squares (`scipy.optimize.curve_fit` (Virtanen et al., 2020)), an increasing and a decreasing form:

$$\hat{U}_{\text{inc}}(z) = c_{\text{off}} + c_{\text{sc}} \frac{z^n}{K_d^n + z^n},$$
$$\hat{U}_{\text{dec}}(z) = c_{\text{off}} + c_{\text{sc}} \frac{K_d^n}{K_d^n + z^n},$$

with parameters $c_{\text{off}} \geq 0$, $c_{\text{sc}} \geq 0$, $K_d > 0$, and $n \in [0.1, 10]$. To down-weight grid points where ensemble estimates are unreliable, fitting is performed with inverse-standard-deviation weighting ($\sigma_i = \hat{s}(z_i)$). The best model is selected by lowest weighted mean squared error.

For the Konrath-based model, the increasing Hill function achieves a substantially lower weighted MSE than the decreasing form and is selected as the best fit:

$$\hat{U}_{\text{Konrath}}(z) = 1.668 + 13.830 \frac{z^{2.114}}{3.245^{2.114} + z^{2.114}}.$$

For the Hunziker-based model, the increasing Hill function is likewise selected:

$$\hat{U}_{\text{Hunziker}}(z) = 0.778 + 0.263 \frac{z^{3.652}}{0.667^{3.652} + z^{3.652}}.$$

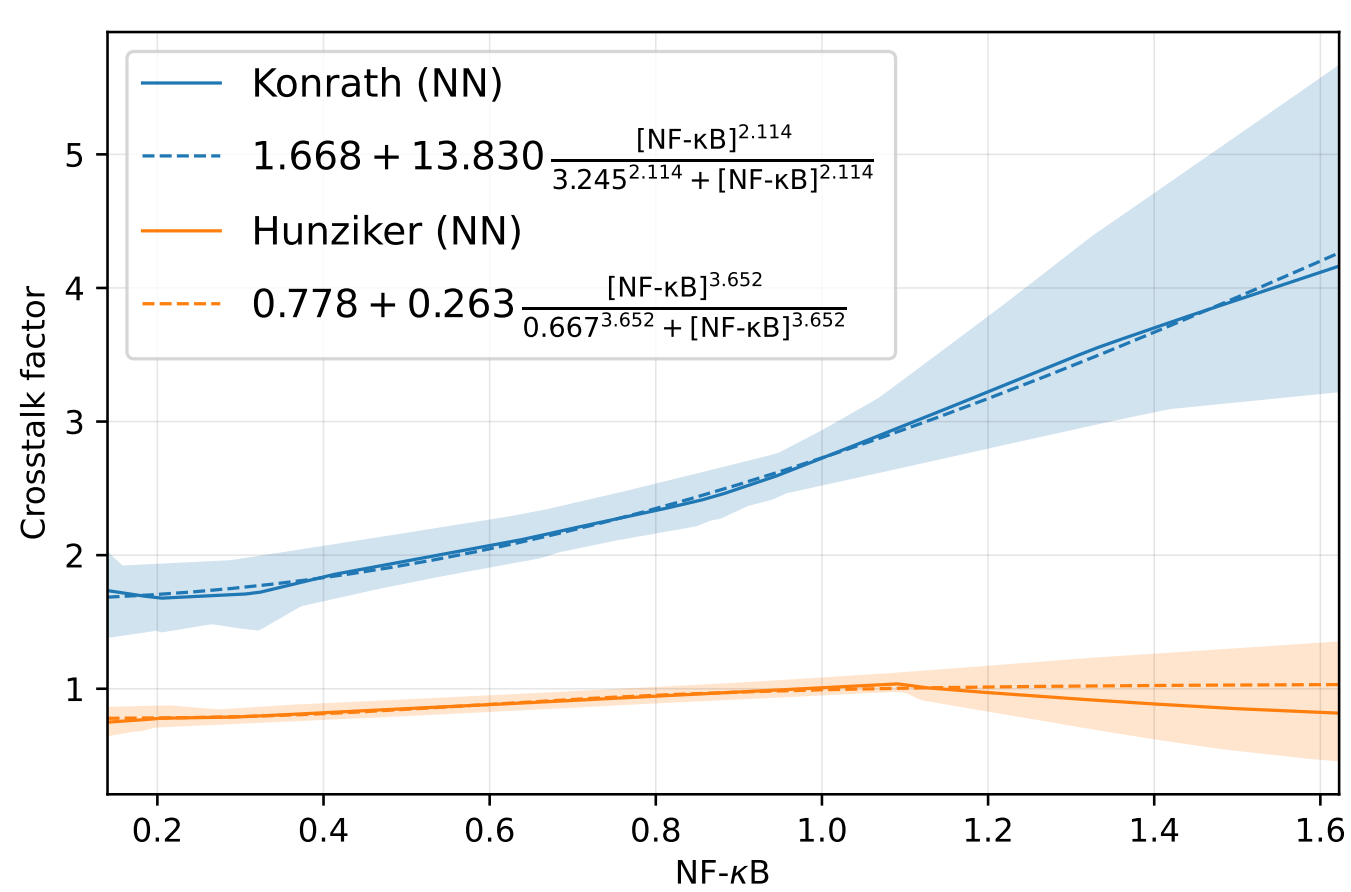

Figure S6: Hill regression fits to the learned crosstalk functions. Solid lines show the ensemble mean of $U_\theta(z)$ across independent training runs; shaded bands indicate the 5th–95th percentile range. Dashed lines show the best-fitting Hill function for each dataset (Konrath in blue, Hunziker in orange).

15.1 Derivation of the series approximation

The Hill regression for the Konrath model yields

$$\hat{U}_{\text{Konrath}}(z) = c_{\text{off}} + c_{\text{sc}} \frac{z^n}{K_d^n + z^n}, \tag{12}$$

with fitted parameters $c_{\text{off}} = 1.668$, $c_{\text{sc}} = 13.830$, $K_d = 3.245$, and $n = 2.114$. We first factor K_d^n from the denominator:

$$\frac{z^n}{K_d^n + z^n} = \frac{z^n}{K_d^n\left(1 + \left(\frac{z}{K_d}\right)^n\right)} = \frac{1}{K_d^n} \cdot \frac{z^n}{1 + \left(\frac{z}{K_d}\right)^n}. \tag{13}$$

Defining $\xi \equiv (z/K_d)^n$, the fraction becomes

$$\frac{z^n}{K_d^n + z^n} = \frac{\xi}{1+\xi} \quad \text{with} \quad \xi = \left(\frac{z}{K_d}\right)^n. \tag{14}$$

Our NF-κB signal satisfies $z < 1.6$, while $K_d = 3.245$, and consequently $|\xi| < 1$. Because $|\xi| < 1$, we may expand $(1+\xi)^{-1}$ as a convergent geometric series:

$$\frac{1}{1+\xi} = \sum_{k=0}^{\infty} (-1)^k \, \xi^k = 1 - \xi + \xi^2 - \xi^3 + \cdots \tag{15}$$

Multiplying both sides by ξ gives

$$\frac{\xi}{1+\xi} = \xi - \xi^2 + \xi^3 - \cdots = \sum_{k=1}^{\infty} (-1)^{k+1} \, \xi^k. \tag{16}$$

Substituting $\xi = (z/K_d)^n$ and retaining only the leading term ($k = 1$):

$$\frac{z^n}{K_d^n + z^n} \approx \left(\frac{z}{K_d}\right)^n = \frac{z^n}{K_d^n}. \tag{17}$$

Inserting the leading-order approximation (17) into the full Hill function (12):

$$\hat{U}_{\text{Konrath}}(z) \approx c_{\text{off}} + c_{\text{sc}} \cdot \frac{z^n}{K_d^n} = c_{\text{off}} + \frac{c_{\text{sc}}}{K_d^n} \, z^n. \tag{18}$$

Evaluating the prefactor numerically:

$$\frac{c_{\text{sc}}}{K_d^n} = \frac{13.830}{3.245^{2.114}} \approx \frac{13.830}{12.047} \approx 1.148. \tag{19}$$

Therefore, the first-order series approximation of the Konrath Hill function in the low-saturation regime is

$$\hat{U}_{\text{Konrath}}(z) \approx 1.668 + 1.148 \cdot z^{2.114}. \tag{20}$$

References

J. Bradbury, R. Frostig, P. Hawkins, M. J. Johnson, C. Leary, D. Maclaurin, G. Necula, A. Paszke, J. VanderPlas, S. Wanderman-Milne, and Q. Zhang. JAX: composable transformations of Python+NumPy programs, 2018. URL http://github.com/jax-ml/jax.

B. R. Brum, L. Lober, I. Previdelli, and F. A. Rodrigues. Discovering equations from data: symbolic regression in dynamical systems. *Journal of Physics: Complexity*, 7(1):012001, feb 2026. doi: 10.1088/2632-072X/ae3eed. URL https://doi.org/10.1088/2632-072X/ae3eed.

S. L. Brunton, J. L. Proctor, and J. N. Kutz. Discovering governing equations from data by sparse identification of nonlinear dynamical systems. *Proceedings of the National Academy of Sciences*, 113(15):3932–3937, Mar. 2016. ISSN 1091-6490. doi: 10.1073/pnas.1517384113. URL http://dx.doi.org/10.1073/pnas.1517384113.

G. Careccia, F. Colombo, M. Tirone, A. Agresti, M. E. Bianchi, S. Zambrano, and E. Vénéreau. Exploiting live imaging to track nuclei during myoblast differentiation and fusion. *Journal of Visualized Experiments*, (146), Apr. 2019. ISSN 1940-087X. doi: 10.3791/58888. URL http://dx.doi.org/10.3791/58888.

E. Colombo, S. Pozzi, A. Loffreda, F. Genova, E. Aloi, T. Heinichen, P. Falletta, M. Mazzocca, T. Fillot, D. Gnani, A. Agresti, M. E. Bianchi, S. Zambrano, and D. Mazza. NF-κB transcriptionally enhances p53 accumulation dynamics hampering DNA repair. bioRxiv 10.64898/2026.02.24.707448. Feb. 2026. doi: 10.64898/2026.02.24.707448. URL http://dx.doi.org/10.64898/2026.02.24.707448.

M. Cranmer. Interpretable machine learning for science with pysr and symbolicregression.jl, 2023. URL https://arxiv.org/abs/2305.01582.

DeepMind, I. Babuschkin, K. Baumli, A. Bell, S. Bhupatiraju, J. Bruce, P. Buchlovsky, D. Budden, T. Cai, A. Clark, I. Danihelka, A. Dedieu, C. Fantacci, J. Godwin, C. Jones, R. Hemsley, T. Hennigan, M. Hessel, S. Hou, S. Kapturowski, T. Keck, I. Kemaev, M. King, M. Kunesch, L. Martens, H. Merzic, V. Mikulik, T. Norman, G. Papamakarios, J. Quan, R. Ring, F. Ruiz, A. Sanchez, L. Sartran, R. Schneider, E. Sezener, S. Spencer, S. Srinivasan, M. Stanojević, W. Stokowiec, L. Wang, G. Zhou, and F. Viola. The DeepMind JAX Ecosystem, 2020. URL http://github.com/google-deepmind.

R. N. Gutenkunst, J. J. Waterfall, F. P. Casey, K. S. Brown, C. R. Myers, and J. P. Sethna. Universally sloppy parameter sensitivities in systems biology models. *PLOS Comp. Bio.*, 3(10):e189, Oct. 2007. ISSN 1553-7358. doi: 10.1371/journal.pcbi.0030189. URL http://dx.doi.org/10.1371/journal.pcbi.0030189.

A. Hunziker, M. H. Jensen, and S. Krishna. Stress-specific response of the p53-mdm2 feedback loop. *BMC Systems Biology*, 4(1), July 2010. ISSN 1752-0509. doi: 10.1186/1752-0509-4-94. URL http://dx.doi.org/10.1186/1752-0509-4-94.

P. Kidger. *On Neural Differential Equations*. PhD thesis, University of Oxford, 2021.

P. Kidger and C. Garcia. Equinox: neural networks in JAX via callable PyTrees and filtered transformations. *Differentiable Programming workshop at Neural Information Processing Systems 2021*, 2021.

F. Konrath, A. Mittermeier, E. Cristiano, J. Wolf, and A. Loewer. A systematic approach to decipher crosstalk in the p53 signaling pathway using single cell dynamics. *PLOS Comp. Bio.*, 16(6):e1007901, June 2020. ISSN 1553-7358. doi: 10.1371/journal.pcbi.1007901. URL http://dx.doi.org/10.1371/journal.pcbi.1007901.

A. Kværnø. Singly diagonally implicit runge–kutta methods with an explicit first stage. *BIT Numerical Mathematics*, 44(3):489–502, 2004.

I. Loshchilov and F. Hutter. Decoupled weight decay regularization. In *International Conference on Learning Representations*, 2019. URL https://openreview.net/forum?id=Bkg6RiCqY7.

G. Massonis, A. F. Villaverde, and J. R. Banga. Distilling identifiable and interpretable dynamic models from biological data. *PLOS Computational Biology*, 19(10):e1011014, Oct. 2023. ISSN 1553-7358. doi: 10.1371/journal.pcbi.1011014. URL `http://dx.doi.org/10.1371/journal.pcbi.1011014`.

V. Nair and G. E. Hinton. Rectified linear units improve restricted boltzmann machines. In J. Fürnkranz and T. Joachims, editors, *Proceedings of the 27th International Conference on Machine Learning (ICML-10), June 21-24, 2010, Haifa, Israel*, pages 807–814. Omnipress, 2010. URL `https://icml.cc/Conferences/2010/papers/432.pdf`.

N. Schmid, D. Fernandes del Pozo, W. Waegeman, and J. Hasenauer. Assessment of uncertainty quantification in universal differential equations. *Philos Trans A Math Phys Eng Sci*, 383(2293), Apr. 2025. ISSN 1471-2962. doi: 10.1098/rsta.2024.0444. URL `http://dx.doi.org/10.1098/rsta.2024.0444`.

G. Söderlind. Digital Filters in Adaptive Time-Stepping. *ACM Transactions on Mathematical Software*, 20 (1):1–26, 2003.

C. Stringer, T. Wang, M. Michaelos, and M. Pachitariu. Cellpose: a generalist algorithm for cellular segmentation. *Nature Methods*, 18(1):100–106, Dec. 2020. ISSN 1548-7105. doi: 10.1038/s41592-020-01018-x. URL `http://dx.doi.org/10.1038/s41592-020-01018-x`.

P. Stumm and A. Walther. New algorithms for optimal online checkpointing. *SIAM Journal on Scientific Computing*, 32(2):836–854, 2010. doi: 10.1137/080742439.

P. Virtanen, R. Gommers, T. E. Oliphant, M. Haberland, T. Reddy, D. Cournapeau, E. Burovski, P. Peterson, W. Weckesser, J. Bright, S. J. van der Walt, M. Brett, J. Wilson, K. J. Millman, N. Mayorov, A. R. J. Nelson, E. Jones, R. Kern, E. Larson, C. J. Carey, İ. Polat, Y. Feng, E. W. Moore, J. VanderPlas, D. Laxalde, J. Perktold, R. Cimrman, I. Henriksen, E. A. Quintero, C. R. Harris, A. M. Archibald, A. H. Ribeiro, F. Pedregosa, P. van Mulbregt, and SciPy 1.0 Contributors. SciPy 1.0: Fundamental Algorithms for Scientific Computing in Python. *Nature Methods*, 17:261–272, 2020. doi: 10.1038/s41592-019-0686-2.

Q. Wang, P. Moin, and G. Iaccarino. Minimal repetition dynamic checkpointing algorithm for unsteady adjoint calculation. *SIAM Journal on Scientific Computing*, 31(4):2549–2567, 2009. doi: 10.1137/080727890.

T. Westny, A. Mohammadi, D. Jung, and E. Frisk. Stability-informed initialization of neural ordinary differential equations. In *ICML Proceedings*, 2024.
